# Comparative Environmental Impacts and Development Benefits of Coastal Aquaculture in Three Tropical Countries: Madagascar, Tanzania and Indonesia

**DOI:** 10.21315/tlsr2023.34.3.15

**Published:** 2023-09-30

**Authors:** Andreas Kunzmann, Gildas Todinanahary, Flower E. Msuya, Yustian Alfiansah

**Affiliations:** 1 Leibniz Centre for Tropical Marine Research, Fahrenheitstraße 6, 28359, Bremen, Germany; 2 Institut Halieutique et des Sciences Marines, BP 141 - Route du Port, Av. De France, Tulear 601, Toliara, Madagascar; 3 Zanzibar Seaweed Cluster Initiative, Malindi Rd, Zanzibar, Tanzania; 4 Alfred-Wegener-Institute for Polar and Marine Research, Am Handelshafen 12, 27570 Bremerhaven, Germany; 5 Research Centre for Oceanography, Indonesian Institute of Sciences, Pasir Putih I, Road. East Ancol North Jakarta 14430 DKI Jakarta 14, Indonesia

**Keywords:** Tropical Coasts, Livelihood Diversification, Aquatic Ecosystems, Marine Pollution, Seafood

## Abstract

Aquaculture is still in early development in Madagascar and Tanzania, while in Indonesia, aquaculture has a long history. In Madagascar, villagers are farming seaweed and sea cucumbers, as part of small-scale community-based aquaculture (CBA). They followed a contractual model between a private farming company and farmers. Local non-governmental organisations (NGOs) and public institutions in Madagascar jointly strive to reverse the trend of ongoing anthropogenic coastal degradation. In Tanzania, the cultivation of red seaweeds has been established for over 30 years, with declining production attributed to climate change. While shrimp farming still involves, to some extent, clearing of mangroves in Tanzania, seaweed culture has only mild impact on coastal ecosystems. Farming areas provide shelter and habitat for juvenile fish, crabs and other organisms. Therefore, NGOs ask for support to improve culture methods. Various problems and shortcomings in Indonesia have been clearly identified, including issues related to new aquaculture areas, pollutants, emerging diseases, insufficient broodstock and fry supply, as well as a lack of technology and manpower. To address these challenges and ensure the growth of aquaculture production, the government has implemented national policies and established training and broodstock centers throughout the country. In Madagascar, the CBA programme stands out as a success story and can serve as a template for other coastal regions and countries. In Tanzania, the adoption of CBA model for co-culture could be the future. In Indonesia, due to a very long coastlines and complicated legislation, IMTA seems to be particularly suitable, as successfully tested in model regions.

**Highlights**

- In Madagascar, villagers followed a contractual model between a private farming company and the farmers.
- In Tanzania, the non-government organisations (NGOs) of farming ask for support to improve culture methods and establish co-culture, ideally integrated multi-trophic aquaculture (IMTA).
- In Indonesia, the government issued new national policies and established training and broodstock-centres all over the country.
- South-south collaboration should be more promoted (e.g., through close scientific collaboration) more, so the three countries can benefit from each other’s experiences.

## INTRODUCTION

Aquaculture is a rapidly growing global industry that feeds the expanding population. Most of the seafoods from aquaculture come from fish followed by seaweed, crustaceans and shellfish. The total aquaculture production comprised 87.5 million tonnes of aquatic animals mostly for human food, while seaweed production reached to 34.7 million tonnes for food as well as other needs such as pharmaceutical industry, feed and cosmetics (FAO 2022; Cai 2021). According to Food and Agricultural Organisation (FAO 2018), seaweed aquaculture is the second-largest product of aquaculture, following fish, and it is a worldwide multimillion-dollar livelihood activity. Moreover, in marine aquaculture (or “mariculture”), finfish cages and crustaceans contribute 23.8% of total marine production (23 million tonnes), while shelled molluscs account for 74.6%. Estuarine or brackish water aquaculture produce shrimps/prawns and finfish in amount of 55.6% and 12.2% of total brackish water aquaculture production (10 million tonnes), respectively (FAO 2022). Tropical aquaculture accounts for over 70% of global aquatic animal and seaweed production, significantly impacting the world’s seafood supply. Nevertheless, it faces challenges due to the lack of sufficient research, effective policies, and environmental sustainability.

Marine aquaculture in tropical countries usually takes place in estuarine and coastal areas. Troell (2009) comprehensively determined marine and brackish-water aquaculture in the tropical countries. The estuarine/brackish-water aquaculture uses various pond systems such as traditional, extensive, semi-intensive and intensive systems, while mariculture in coastal areas utilises pens, cages, stakes, rafts, longlines and beds. Mariculture commodities contribute significantly to the income of the developing counties, such as Madagascar, Tanzania and Indonesia. Madagascar produces seaweeds and sea cucumbers as their main aquaculture products, while in Tanzania seaweeds dominate. Tanzania is known as the third largest producer of *Eucheuma denticulatum* after Indonesia and Philippines. Indonesian brackish- and marine water aquaculture implement various species, production facilities and methods. The most important commodities in 2019 (Kementerian Kelautan dan Perikanan [KKP] 2020) were seaweed (*E. denticulatum*), crustaceans (*Penaeus vannamei* and *P. monodon*), fish (milkfish and barramundi) and shellfish.

Given the impact of climate change, increased pollution in coastal areas, and potential socio-economic conflicts that can threaten coastal livelihoods and aquaculture productivity in tropical countries, it is necessary to review both good aquaculture practices and challenges in these regions. Madagascar, Tanzania and Indonesia are among tropical countries which can be chosen as models to develop, maintain and increase the aquaculture productivities. As two tropical countries in Africa, Madagascar and Tanzania focus their mariculture on exotic species such as holothurians and seaweed. Among all countries in Southeast Asia, Indonesia has the widest area (both potential and active areas) for mariculture with diverse cultured biotas.

This review summarises lessons and experiences, but also bottlenecks, shortcomings and potential solutions for the future from several years of research and development in the three tropical countries. While in Madagascar, the focus is on community-based poly-aquaculture of sea cucumber, seaweed and corals, seaweed aquaculture is the main commodity in Tanzania; and seaweed, shrimp, and fish dominate aquaculture practice in Indonesia.

## METHODOLOGY

We collected information from scientific articles, government policy papers, series of regulations and laws from open access platforms, including journals, conference proceedings and IOP-Science, as well as official ministerial websites, written by local or international scientists in national languages (i.e., Indonesian and French language) as well as in English. We used terminologies which are common for the three regions, such as “marine aquaculture”, “environmental and ecological impact” and “main cultured biota”. After identifying and describing problems in each country/region, we offer solutions based on existing results, regulation and law as lesson learned.

## RESULTS AND DISCUSSIONS

### Lessons Learned from The Development of Community-Based Poly-Aquaculture in Madagascar

#### General aquaculture development

Community-based aquacultures (CBAs) are the most important community-scale aquaculture activities that exist in Madagascar, particularly promoted in the coastal villages of the Southwest region alongside with the industrial aquaculture of shrimps and eels in the Northwest and Southeast (Tsiresy *et al*. 2011; Todinanahary *et al*. 2016). Developed for a decade, sea cucumber and seaweed farming are among the most promising alternative sources of income for coastal communities. Currently, more than 40 villages off the ~300 km coastline are farming sea cucumber (*Holothuria scabra*) and/or seaweed (*Kappaphycus alvarezii*) for commercial purposes (Eeckhaut *et al*. 2008; Lavitra 2018). In 2020, the production of sea cucumbers from communities’ farms reached more than 135,000 adult holothurians corresponding to 2.4 tonnes of dry trepang (Indian Ocean Trepang 2020, https://www.iotrepang.com/info). The production of seaweed from about 1,600 farmers reached 1,542 tonnes dry weight (Ocean Farmers 2020). To date, less than 100 sea cucumber farmers and nearly 2,000 seaweed farmers are operating. On nearly 250 km of the southwest coastline, a total of 317 ha of suitable sites for sea cucumber farming have been identified, but only 3.7% of this area are used. Suitable sites for seaweed farming amount to 4,427 ha, of which less than 20% are used (Mollion 2020).

The production system for CBA follows a contractual model described in Todinanahary *et al*. (2016), between a private farming company and each farmer, both forming the production unit. The farming company ensures the sustainability of the production, technical support and investment in production materials, while the farmers manage their farm plot by following the farming protocols (Todinanahary *et al*. 2016).

For seaweed farming, the main environmental risks are the development of the Epiphytic Filamentous Algae (EFA) (Tsiresy *et al*. 2016), hyper-sedimentation due to coastal erosion, desalination of the farm waters during periods of heavy rain or river flooding, and devastation of algae fields through tropical storms (Todinanahary *et al*. 2016). These risks may also affect the sea cucumber farming, considering that sea pens are located in shallow waters, at the intertidal zone. In addition to this, the predation by crabs and the Skin Ulceration Disease also impacts the survival and production rate of *Holothuria scabra* farming (Eeckhaut *et al*. 2020).

The continuous development of these activities combined with the saturation of suitable spaces can contribute to the disruption of fragile ecosystems, in particular coral reefs. Indeed, farmers, increasing their production lines, sometimes create a clearing in the middle of corals to implement their new seaweed lines (pers. observation of the authors). The sustainability of CBA relies on the diversification of cultivated species, while respecting the natural ecosystems in the installation area. The development of CBA for the future should be oriented towards poly-aquaculture or IMTA in order to increase economic, environmental and social benefits.

#### Environmental and ecological impacts of community-based aquaculture: Focus on plastic waste

In 2020, Rabemanantsoa (2021) assessed the environmental impacts of plastic waste from fishing activities, seaweed and sea cucumber farming in one of the most productive areas of Madagascar for CBA. In this area, plastics are used to fabric fishing nets, pots and traps. For seaweed farming, they are used particularly for the whole farming structures such as the main line, loops and branching lines, floats, harvesting boats and drying racks. For the sea cucumber industry, they are intended for use in grids to build sea pens. All these activities use plastic for economic, technical and practical reasons.

The most obvious impact is the dispersion of plastic waste into the environment, leading to environmental pollution (Ganguly *et al*. 2018; Rabemanantsoa 2021). More than 1,000 plastic waste pieces weighing almost 15 tonnes was collected at the beaches, on a total area of only 6,000 m^2^. Fishing is the source of 30% of the plastic waste, which is mainly composed of ropes, pieces of nets, sandals or floats, mosquito nets, thread and pieces of plastic bottles. The remainder is attributed to daily consumables including plastic bags and packaging. The sea cucumber farming rarely generates plastic waste.

**Figure 1:**
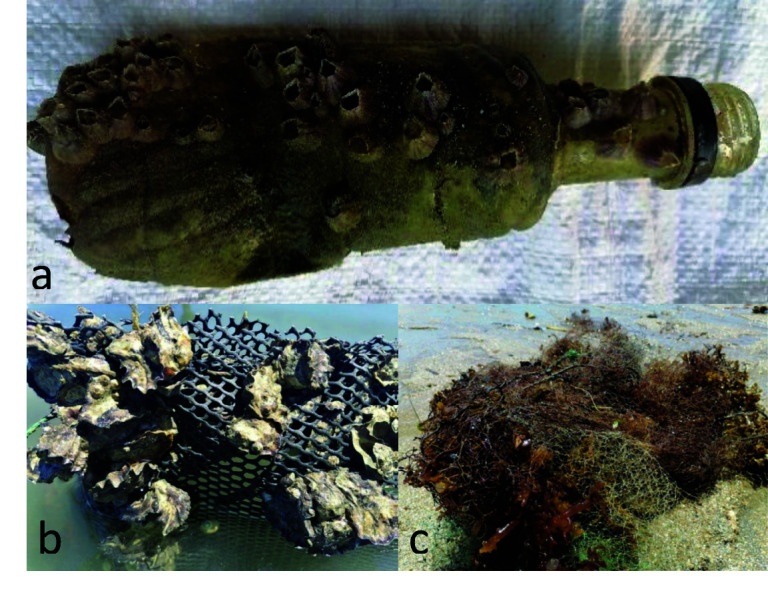
Observed wasted plastic materials from aquaculture villages. (a) Domestic plastic bottle colonised by barnacles; (b) HDPE seapen enclosure colonised by Crassostrea species; and (c) Fishing net having torn off live corals and cultured algae, observed during the landing of fishermen on the beach (modified from Rabemanantsoa 2021).

The plastic pollution of these sources contaminates fragile ecosystems, e.g., the fringing coral reef off Sarodrano (Fig. 1). These waste cover reef habitats and benthic communities, plug cavities, and damage and tear benthic species such as sponges, algae and corals. In addition, drifting plastic waste also makes excellent dissemination media to various species that can be invasive such as barnacles and tube worms (Rabemanantsoa 2021). Plastic waste from aquaculture is not observed in abundance on the reef ecosystem. This can be explained by the fact that plastic waste from aquaculture, especially seaweed is mainly washed up on the beach, where farming materials are thrown away by farmers after they prepare the cuttings and finish harvesting the farmed seaweed (Todinanahary *et al*. 2016).

The deposits of waste on the beaches reflect the mismanagement with used plastics. Due to the lack of garbage bins and waste disposal structures, incineration is the “normal” way to dispose of this bulky waste in the village of Sarodrano.

#### Conclusions and future perspectives for Madagascar

To overcome the environmental risks and impacts of the CBA, it is necessary to strengthen measures to raise awareness and educate the population on the destruction of habitats and other dangers that plastic waste can bring. Cooperation between the existing operating sectors in the fishing villages and the fishermen on the establishment of a treatment and recovery plant for waste from fishing and aquaculture is needed. It would also be wise to develop inexpensive strategies to monitor and assess marine litter generated by the fishing and village aquaculture sectors. Moreover, using coral farming as educative and restorative solution could constitute one of the promising solutions to indirectly overcome the environmental and ecological problems generated by the CBA. Indeed, several studies report the importance of the CBAs and their impacts on the coral reefs, which represent the basis of the income-generating activities for the coastal communities of Madagascar (Eeckhaut *et al*. 2008; Todinanahary *et al*. 2016; Lavitra 2018).

Since 2013, several activities involving coral breeding, establishment of nurseries, coral gardens and artificial reefs have been undertaken by and with stakeholders. Research from the region showed that small scale CBA of corals is technically feasible and environmentally safe (Todinanahary *et al*. 2017). Moreover, it was shown that the used techniques (e.g., nurseries, coral gardens, artificial reefs) are simple, low-cost and can be profitable and suitable to coastal communities with satisfactory economic results (Todinanahary *et al*. 2017). Furthermore, coral farming is also used for restoration and reef conservation programmes, directly involving and benefitting the local communities.

However, community-based coral farming will only be able to develop and fully accepted if the communities see a financial interest in it. As with seaweed and sea cucumbers, a decrease in economic yield is often accompanied by an abandonment of aquaculture and a return towards full time traditional fishing. Economically, Todinanahary *et al*. (2017) demonstrated the feasibility of small-scale village coralliculture that fits very well into the overall development scheme of community-based poly-aquaculture of seaweed and sea cucumbers.

The co-existence of all the above-mentioned forms of aquaculture forms the basis of a successful and sustainable CBA and can contribute to reducing the pressure on the marine environment in order to secure food and jobs for villagers. For the private companies involved in the process, the human potential, and diversified marine resources are readily available. NGOs (e.g., Blue Ventures and Wildlife Conservation Society [WCS]), local associations for community management (e.g., Velondriake and Soariake) and research centres [e.g., University of Toliara and Institut Halieutique et des Sciences Marines (IH.SM)] should support village aquaculture projects through joint programmes.

**Figure 2:**
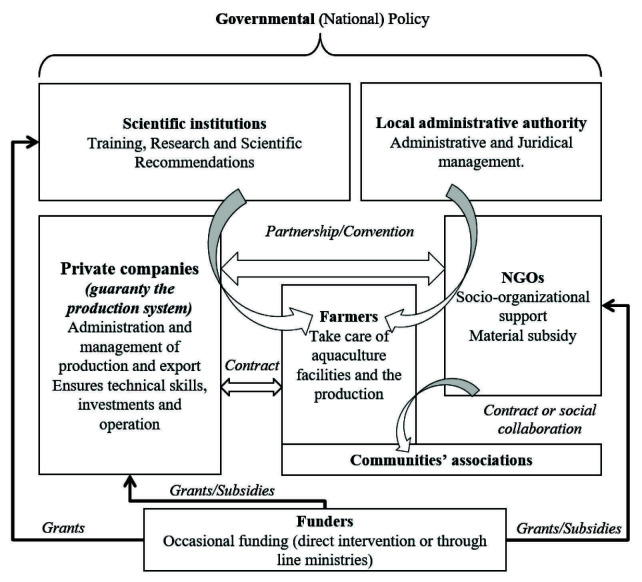
Good organisational practice in community-based aquaculture (adapted from Todinanahary *et al*. 2016).

The CBA is presently developing with a promising sustainable governance, involving all the actors (communities, private companies, civil society, public institutions), who are meeting in a quarterly discussion platform (Fig. 2). With 10 years experiences, it remains the most promising alternative to declining coastal fishery and a tool for coastal community resilience. Coastal communities and private farming companies constitute the main producers (notably of seaweed and sea cucumber), while NGOs are supporting social organisation and public institutions oversee administrative (Fishery Administration) and research (IH.SM) purposes. This type of model should be encouraged.

**Figure 3:**
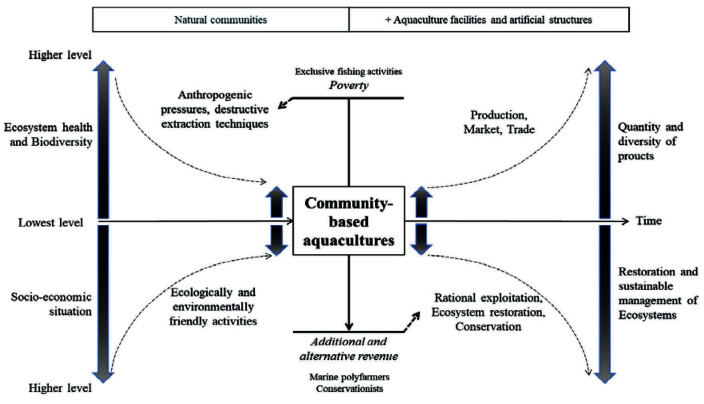
Bio-ecological and socio-economic interests of community-based aquaculture. Grey: high level, Black: critical level (adapted from Todinanahary 2016).

An optimistic view into the future, foresees the potential nationwide application of the lessons learned from the CBA in coastal villages of southwestern Madagascar. This could reverse the trend of ongoing degradation of coral reefs and adjacent ecosystems as illustrated in Fig. 3.

Changing the habitude of these fishermen by offering them alternative means to traditional fishing would reduce these pressures if the practices of CBA were to become widespread. Various economic outlets (e.g., restoration programme, development of products used in medicine), could create a market for the sale of corals and promote the repopulation of reefs.

### Lessons Learned from Seaweed Aquaculture in Tanzania

#### Seaweed aquaculture development and environment in Tanzania

**Figure 4:**
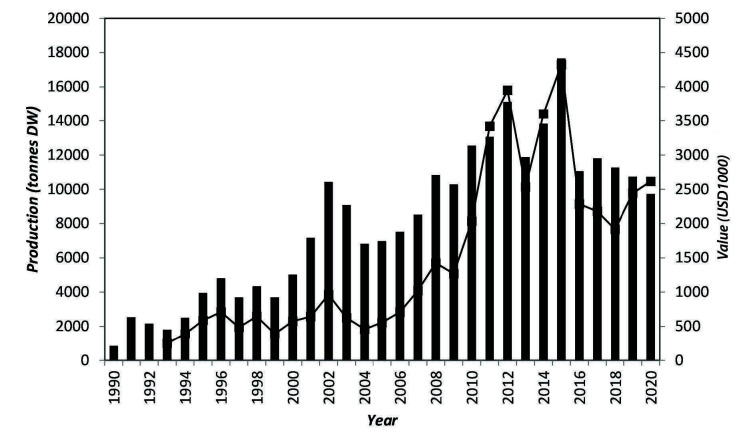
Seaweed production in Tanzania, 1990–2020.

In Tanzania, the red seaweeds *Eucheuma* and *Kappaphycus* have been cultivated for over 30 years employing about 30,000 farmers as a major livelihood. Production in recent years has decreased from above 16,000 tonnes (USD4 million) in 2015 to the current 11,000 tonnes (USD2 million) in 2020 (See Fig. 4). The fall in production is attributed to climate change impacts causing ice-ice disease and epiphytes (Msuya & Porter 2014; Largo *et al*. 2020). Additionally, low prices paid to farmers and the decrease in seaweed production discourage farmers, some of whom have stopped farming causing further decrease in the production (Msuya & Porter 2014; Largo *et al*. 2020).

Like in other countries worldwide, aquaculture of seaweed in Tanzania has milder impact on the environment compared with, e.g., fish culture. However, placing seaweed farms in an area affects seagrass and macroalgae cover and associated organisms, affecting ecosystem services provided by such plants and animals. Seaweed farming causes disturbances to the sediment and organisms through trampling, shading and mechanical abrasion of the sediment by the seaweed lines (De La Torre-Castro & Rönnbäck 2004; Moreira-Saporiti *et al*. 2021). Msuya *et al*. (1996) showed lower percent covers of macrophytes and macro-benthos in seaweed farms compared with areas where seaweed was not farmed. Similarly, Johnstone and Olafsson (1995) and Ólafsson *et al*. (1995) observed lower numbers of meiofauna in seaweed compared to non-seaweed farms. Brown and Taylor (1999) showed a 50% reduction in macro-benthos densities following experimental trampling and Lyimo *et al*. (2006) reported lower biomass of seagrasses in seaweed than non-seaweed farming sites. In a more recent work, Moreira-Saporiti *et al*. (2021) found that trampling reduced percent cover of seagrasses and macroalgae. On deforestation, Msuya *et al*. (2007) and Msuya (2013) showed that some pegs (sticks) used for farming seaweed were from mangroves.

None of these studies showed severe impact of seaweed farming on the environment, but rather benign impacts, whereas other forms of aquaculture such as fish and shrimp farming involve clearing of mangroves for pond construction (Ateweberhan *et al*. 2018). However, it is important to use conservation measures to reduce possible larger scale impacts including selecting sites without rich seagrass, algae and sessile animals, and replanting/replenishing the marine environment. In the next section we give details of specific impacts that seaweed farming can have on the environment.

#### Impact on seagrasses, macroalgae and macro-benthos

Msuya *et al*. (1996) conducted the first study of environmental impact on effect of seaweed farming on macrophytes (macroalgae and seagrasses) in Zanzibar, Tanzania. This study showed that 174 and 255 quadrats had partial seagrass cover in the seaweed farming and non-seaweed farming areas, respectively. Those with seagrass cover above 70% were 88 quadrats in the seaweed farming sites and 118 in the non-seaweed farming sites. Likewise, Lyimo *et al*. (2006) and Lyimo *et al*. (2009) found lower seagrass biomass in seaweed farming sites compared with non-seaweed farming sites. Thus, it can be concluded that reduction in seagrass cover is associated with seaweed farming activities.

For macroalgae on the other hand, the authors showed that seaweed farming sites had more algae cover compared to the non-seaweed sites. This may show that more algae started growing in the seaweed farming areas, attracted by changes in ecosystems (such as shading, trampling and abrasion) following the introduction of seaweed farming in the areas. However, the algae were found in areas from the beach to about 20 m before the seaweed farms, while in the farms few or no algae were observed. This may be due to competition between the farmed seaweed and other algae. Moreira-Saporiti *et al*. (2021) found out that where seagrasses were decreasing, opportunistic macroalgae increased. The lower percent cover by algae recorded by Msuya *et al*. (1996) at the lower intertidal areas where seaweed farms are located compared to the upper intertidal areas indicates a result of shading of the seagrasses and algae by the overlying farmed seaweed, reducing the amount of sunlight needed for growth.

In macrobenthos studies, sea urchins (one of the main grazers of the farmed seaweed) and starfish (Ophioroids) were rarely found in seaweed farming sites, while tubeworms and crabs were more abundant in the seaweed farming sites (Msuya *et al*. 1996). Tubeworms used dead seagrass roots as shelters by making tubes out of the dead seagrass roots. These results show that seaweed farming attracts opportunistic organisms such as algae and tubeworms. The presence of tubeworms may be considered a succession process; where seagrasses are dying, opportunistic tubeworms use seagrass roots as shelters. Similarly, Lyimo *et al*. (2009) found lower diversity and abundance of macro-benthos in seaweed farming sites compared with non-seaweed sites. Krishnakumar (2003) reported that the death of seagrasses encourages tubeworms, and that farmers also remove associated macro-benthic organisms causing lower abundances. On fish abundances, Msuya *et al*. (1996) showed that seaweed farming areas are also nursery and grow-out grounds where they act as habitats and shelters for juvenile fish, and that farmers get some fish catch that they use for food at home. Similar results were reported by Eklöf *et al*. (2006) who found higher fish catches in traps placed in seaweed farms.

#### Impact on microorganisms and underlying sediment

Seaweed farming causes lower meio-benthos counts in seaweed farming sites. Ólafsson *et al*. (1995) found differences in the abundance, composition, and assemblage structures of meio-benthos in seaweed farming and non-seaweed sites with reduced meiofauna abundance and total nitrogen concentrations in seaweed farming sites compared to sites far from the farms.

Msuya *et al*. (1996) pointed out that seaweed farming can also have an effect on the rate of shore erosion or accretion and particle size of the sediment. The authors found more sand (percent composition), less mud and less broken shells in the seaweed farming compared to non-seaweed sites, respectively. Ólafsson *et al*. (1995) reported smaller sediment particles in seaweed farming sites than non-seaweed farming sites. The lower percentage of mud in seaweed farming sites may be caused by wading by farmers, resuspending the mud which is carried away by ocean waves leaving behind coarse sand. It could also be that farmers place seaweed farms in sandy areas rather than muddy areas so as to make fixing of pegs easier (Msuya *et al*. 1996). Krishnakumar (2003) pointed out that farmed seaweed scrapes the substrate surface altering the structure of the sediment and, thus, associated algal mats.

#### Conclusions and future perspectives for Tanzania

The placement of seaweed farms in intertidal areas, which is a habitat for naturally occurring seagrasses, algae and sessile animals, alters the composition and functioning of the ecosystems located in those areas. Seagrasses and algae are among the most important vegetations of the intertidal areas and also among the most productive. The main impact of seaweed farming could be more from physical disturbances such as trampling and abrasion. Lyimo *et al*. (2006) stated that generally seaweed farming does not seem to have direct effects on growth of seagrasses and, therefore, low biomass observed in seaweed farming sites is likely owing to physical disturbances. Moreira-Saporiti *et al*. (2021) stated that seaweed farming impacts seagrasses through trampling and shading, which may in turn cause disturbance to associated organisms such as fish, shelled animals and invertebrates. Equally disturbed will be bacterial mats that are important in nitrogen fixation. In the studies reported here, lower numbers of macro-benthos were recorded in seaweed farming sites. However, all the studies have shown that there is mild impact from seaweed farming considering the partly significant, partly non-significant results obtained in these studies. Eklöf *et al*. (2006) and Moreira-Saporiti *et al*. (2021) for example both showed that some seagrasses, e.g., *Thalassia hemprichii* were little affected by short-time experimental seaweed farming. Seaweed farmers knowledge also contributes to these observations. De La Torre-Castro and Rönnbäck (2004) showed that only 40% of farmers declared that the impact of seaweed farming was a threat to the seagrasses. This is why government, local scientists and NGOs jointly support improved cultivation methods, in order to secure the livelihood of more than 30,000 farmers while conserving the environment. These improved methods include farming in deeper waters, which has enabled the farming of the higher valued *Kappaphycus* and resulted in an increase in general seaweed production (Msuya 2011, 2017; Brugere *et al*. 2020). Co-culture with sea cucumbers and the adoption of a CBA model like in Madagascar could support further increase in production.

### Lessons Learned from Marine and Brackish Water Aquaculture in Indonesia

#### Aquaculture development in Indonesia

Laird (2001) described brackish water aquaculture as a part of mariculture. However, since Indonesian brackish water makes up a greater portion than marine aquaculture, it was attempted to investigate environmental impact separately and identify possible integration of these two mariculture practices. Indonesian marine aquaculture mainly produces fish (grouper and barramundi/seabass) and seaweed (*Kappaphycus* and *Eucheuma*), while coastal aquaculture is usually performed in ponds, so-called “tambak” with saline tilapia (*Oreochromis niloticus*), milkfish (*Chanos chanos*), shrimps (*Penaeus monodon* and *P. vannamei*) and seaweeds (*Caulerpa* sp. and *Gracilaria verrucosa*), which are reared at salinities of 15–30 PSU (KKP 2020).

In this context a number of problems and shortcomings are identified and tackled with different strategies by the Government of Indonesia (GoI). Ecological impacts of intense aquaculture have been reported previously (Phillips *et al*. 2015; Henriksson *et al*. 2019). Common problems include diseases, broodstock and seed availability and lack of technology and manpower. In addition, we attempt to identify specific problems, which have occurred in coastal and mariculture, based on cultured biota, in order to share knowledge with other aquaculturists and countries.

**Figure 5:**
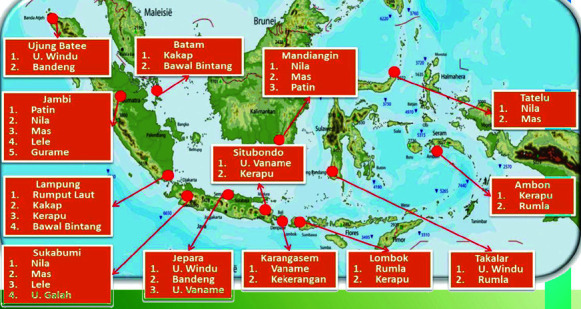
Indonesian centres for broodstocks and seeds/hatcheries (MMAF 2019). “Udang windu”: tiger shrimp (*Penaeus monodon*); “bandeng” = milk fish (*Chanos chanos*); “patin” (*Pangasius* spp.); “ikan mas” (*Cyprinus carpio*), “lele” (*Clarias* spp.); “gurami” (*Osphronemus goramy*); “rumla” = Rumput laut (seaweed); “kerapu” = grouper (*Chromileptes* spp.); “kakap” = barramundi (*Lates calcacifer*); “bawal bintang” = pomfret (Fam. Bramidae); “nila” (*Oreochromis niloticus*); “udang galah” (*Macrobrachium rosenbergii*); and “udang vanname” (*Penaeus vannamei*). Headings of each box refer to local regions.

1. Diseases: Mariculture and coastal aquaculture in Indonesia have to deal with both endemic and sporadic, but devastating disease outbreaks. For example, pacific white-leg shrimp aquaculture in Java Island and Lampung Province suffered from white faeces disease (Alfiansah *et al*. 2020; KKP 2020). In addition, viral disease such as infectious mionecrosis (IMN), Taura syndrome (TS), and white spot syndrome (WSS) occurred occasionally in almost all shrimp aquaculture facilities (tiger and pacific white-leg shrimps) in Indonesia (Taslihan *et al*. 2013; Rahma *et al*. 2014), causing significant economic losses (Taslihan *et al*. 2013). Fish cultured in marine cages also suffered diseases, mainly caused by parasites and bacteria (Herfiani *et al*. 2010; Palm *et al*. 2011; Hennersdorf *et al*. 2016), however, without massive mortalities. In contrast, ice-ice disease caused by pathogenic bacteria massively infected seaweed *Kappaphycus alvarezii* (Achmad *et al*. 2016; Syafitri *et al*. 2017). The disease occurrences strongly correlate with the presence of pathogenic microorganisms, different culture management such as feeding regime, culture densities or with environmental change (i.e., seasonal change) or drastic water quality change including physical and biological parameters, as well as inorganic nutrient concentrations (Palm *et al*. 2011; Syafitri *et al*. 2017; Maryunus 2018; Alfiansah *et al*. 2020).
2. Broodstock and fries/seeds: Demand for seeds of fish, crustaceans and seaweed is high and faster than the growth rate of broodstock. As a result, the use of local broodstock from shrimp culture and catches of wild juveniles from nature (fish and crustaceans) is common. Local seeds are also used for sea weed aquaculture (Hadifa *et al*. 2017), with a drastic decrease during the occurrence of the ice-ice disease. Indonesian shrimp aquaculture also experienced massive disease outbreaks, although a devastating disease like early mortality syndrome/acute hepatopancreatic necrosis disease (EMS/AHPND) has not been detected (Direktorat Jenderal Perikanan Budidaya [DJPB-KKP] 2019). It is assumed that those massive disease outbreaks were caused by the utilisation of brood-stock from shrimp ponds. Consequently, a circular letter was issued, prohibiting employment of mature shrimps from ponds as brood-stocks and establish brood-stock centres in several areas (Fig. 5).
3. Technology and manpower: Indonesian aquacultures usually use a low/experience-based technology approach, with local knowledge transfer among aquaculture operators/technicians, but involve a high amount of manpower. In 2019, aquaculture directly employed about 2.5 million people, with the majority working with freshwater ponds (73%), while mariculture and brackish water ponds employed 12% and 15.5 %, respectively (KKP 2019).

Shrimp aquacultures may become a pioneer for the applications of a medium or high level of technology in Indonesia. This includes recirculating aquaculture system (RAS), application of probiotics and inorganic nutrient reducing agents, which have been applied in hatcheries and grow out facilities of shrimp and fish farming (Suantika *et al*. 2018; Atjo 2019). Moreover, some start-ups in aquaculture have applied digitalisation technology such as automatic feeder, more efficient paddle wheels or nanobubble devices to improve dissolved oxygen concentrations, and also *in-situ* real-time water monitoring. These so-called millennial shrimp farming (MSF) are able to increase productivity, reduce feed conversion ratio and re-use water resources (Bulkini 2021). Thus, pollution through regular effluent discharge, as well as excess of feed supply can be avoided. Moreover, fresh feeds (i.e., low-cost fish, trash fish) have been replaced with formulated feed pellets leading to the establishment of more than 40 feed mills producing shrimp and freshwater and marine fish feed pellets (KKP 2020). Compared to pellet feed, the use of trash fish leads to a much higher feed waste (Hansen *et al*. 1990). However, good quality of pellet feed including those containing substituted essential elements is still under development in terms of their stability and sinking rates, nutrient contents, palatability and efficacy to gain shrimp or fish biomass (KKP 2020).

In the future, Indonesian aquaculture stakeholders need to lower the carbon foot-prints caused by aquaculture activities. Kauffman *et al*. (2017) and Henriksson *et al*. (2018) reported that shrimp aquaculture releases significant amounts of carbon to the environment, mainly due to the use of diesel power, but also the deforestation of mangrove ecosystems and idling opened coastal areas, which were formerly used for aquaculture.

#### Specific problems linked to important aquaculture commodities

Land conversion frequently takes place in coastal ecosystems by cutting mangrove forests. Shrimp farming has resulted in extensive ecological degradation. Indonesia has worldwide the highest level of global mangrove forest loss (45%) during the 21st century (Hamilton & Casey 2014). The estimated mangrove forest cover in Indonesia in 2000 was 24,073.13 km^2^, of which 748.84 km^2^ (about 3.1%) were lost until 2012 (Hamilton & Casey 2014). This equals about 0.26% loss per year between 2000 and 2012 and is substantially lower than in earlier periods such as the 1980s to 2000s (Hamilton 2013).

Water quality deterioration often occurs due to excessive feed input, high organic loaded effluents, disposal of organic and domestic waste to coastal water bodies, and riverine input. This leads to high concentrations of inorganic nutrients, causing eutrophication and increases of anoxic areas due to high biological oxygen demand. In intensive fish cage aquaculture areas mass mortalities of fishes almost happen annually. Greater Jakarta is the most polluted coastal area with very diverse types of pollutants (Dsikowitzky *et al*. 2017; Kunzmann *et al*. 2018; Cordova & Nurhati 2019). Dense fish cages in East Lampung and Pangandaran provoke the deposit of excessive feed, which then leads to nutrient enrichment, massive blooms of phytoplankton and oxygen depletion (Sidabutar 2016). Moreover, harmful dinoflagellates were found in Lampung Bay, where heavy eutrophication occurred (Thoha *et al*. 2019).

The majority of fish farms (i.e., grouper, milkfish and barramundi) use trash fish as the main feed source. Using whole fish is problematic because of its short shelf-life, fluctuating availability (Sim *et al*. 2005) and the fact that it diverts a source of nutritionally-rich food away from direct human consumption domestically (Buchary 2010). Moreover, low-cost fish generally results in poor FCRs, as it breaks up and is partially lost from cages (Sim *et al*. 2005), resulting in eutrophication, development of anoxic sediments, production of hydrogen sulphide, elimination/decrease in benthos (Wu *et al*. 1994), and transmission of parasites (Rückert *et al*. 2009).

Improved compositions of feed ingredients and a combination of improved genetic strains, better environments, water monitoring, feeding practices and best farm management practices can improve FCR values (Ullman *et al*. 2019). Henriksson *et al*. (2019) assumed that FCR for the Pacific white-leg shrimps could be lowered by 20% to 1.16 by 2030, when using good quality feeds pellets. The FCR for fish in most Indonesian aquacultures (i.e., grouper, milkfish and barramundi) is still relatively high (above 5.16) due to the use of trash fish as main feed (Harahap *et al*. 2019). Thus, the usage of crumble and feed pellet is highly necessary to replace trash fish in order to lower FCR (Soemarjati *et al*. 2013; Nuraini *et al*. 2020). The reported FCRs for groupers being fed formulated feeds range between 1.5 and 3.1 (Sim *et al*. 2005; Bunlipatanon *et al*. 2014). From this range, Henriksson *et al*. (2019) assumed that groupers could be entirely fed by pelleted feeds at an FCR of 2 by 2030. Even though these pelleted feeds contain 60% fishmeal and fish oil, and roughly 5 kg fish are needed per kg fishmeal (Parker & Tyedmers 2012), a reduction of 16 kg wild fish per kg grouper may be achieved (Henriksson *et al*. 2019).

#### Strategies to improve aquaculture production in Indonesia

In order to increase aquaculture production, the GoI through the newly created Ministry of Marine Affairs and Fisheries (MMAF/KKP) issued a number of national policies and priorities. These are implemented simultaneously by all stakeholders, including regional governments, private sector, or individuals/aquaculture associations. In addition, MMAF/KKP issued guidelines of good aquaculture practices.

The MMAF provides monitoring services of residual chemicals, biological materials and contaminants in fish farming, not only for aquaculture products, but also for samples from farms/ponds and sea cages, such as water, sediment, and feed pellets. It also recommends shrimp pond settings, regulating broodstocks and hatcheries, and determines strategies to avoid EMS/AHPND outbreak. In addition, high pollution which may occur from shrimp pond effluent discharge (Herbeck *et al*. 2013) can be minimised. Furthermore, the MMAF determines centres or new locations for aquaculture to attract aquaculture investors, as well as empowerment of local people. Also, MMAF applies a combination of aquaculture and re-stocking to natural habitats to implement the Indonesian Biodiversity Strategy and Action Plan 2015–2020. They established show cases for holothurians and lobsters (with minimum size regulations) and oblige aquaculture businesses (individuals, association and industry) to release 2% of harvested lobsters back to nature, while for holothurians, re-stocking with hatchery-produced juveniles is required. With regard to socio-economic sectors, they give an insurance and incentive to small fishermen/aquaculturists to assure their business since 2016.

Demand for good quality seeds is high all over the country, but due to centralised production of seeds, aquaculturists, who run their business far from Java Island have difficulties to access good quality seeds. Therefore, MMAF establishes local technical implementation units to produce the seeds (Fig. 5). This strategy will reduce operational costs and expenses, because aquaculture entrepreneurs will have easy access to good local quality seeds.

#### Conclusions and future perspectives for Indonesia

For the future development of Indonesian aquaculture three main steps for improvement, considering environmental as well as socio-economic aspects, are recommended:

1. Sustainable integrated or polyculture of milk fish, shrimps and seaweed: The future is in sustainable intensification of polyculture, for example by co-culturing milkfish, shrimps, holothurians and seaweed (Contreras-Sillero *et al*. 2020). Alternatively, a sequential integration/partitioned aquaculture system, as proposed by Troell (2009) can be implemented. This farming method allows to flow effluent water containing rich particulate organic matter and inorganic nutrient from shrimp ponds to ponds containing herbivorous fish only, such as milkfish, rabbitfish or saline tilapia, or in polyculture with seaweed and holothurians. This method may lead to (i) an increase and diversification of aquaculture products; (ii) avoidance or minimisation of economic loss due to shrimp diseases; (iii) decreased operational cost for feed pellets (plankton production in ponds); and (iv) optimisation of the usage of space/land without sacrificing existing ecosystems. However, further studies are still needed, especially in terms of proportion of culture biota, precise demand of feed and rearing techniques.
2. Potential application of renewable energy: Aquaculture farms usually depend on electricity supplied by the State Electricity Company or diesel generators. This may lead to high costs and considerable carbon footprint. Thus, the development of renewable energy for aquaculture purposes may become a promising option. Indonesia is the third-largest producer of electricity from geothermal power plants and holds an estimated 40% of the world’s geothermal potential, with an equivalent of 28,000 megawatts (Hasan *et al*. 2012; Semedi *et al*. 2017).
3. Integrated Multi-trophic Aquaculture (IMTA): With 81,000 km coastlines, IMTA seems to be particularly suitable for Indonesia. Here, several species of different trophic levels are cultivated together to optimise the use of nutrients (Buschmann *et al*. 2009). In an ideal case, farmers cultivate fed species such as fish (or shrimps), with detritivores such sea cucumbers or crustaceans (lobster or crabs) grown in cages, together with inorganic nutrient absorbers such as seaweed or mussels. Astriana (2012) reported that this was successfully tested in Nusa Tenggara Barat, by growing oysters (*Crassostrea* sp.), seaweeds (*Gracilaria* sp.) and black tiger shrimps (*Penaeus monodon*). The IMTA can be performed in coastal areas (floating cage aquaculture) or in pond systems. However, we underline that the implementation of IMTA in Indonesia needs substantial political support from several ministries in order to make decisions on locations (spatial planning), essential and high economic cultured biota, and supporting technology and manpower.

Traditional Indonesian aquaculture systems, which want to prepare for the future, have to deal with environmental and ecological problems as mentioned in this review. Particularly the contribution to significant amounts of gas emissions and global warming, due to deforestation of mangroves, abandoned non-productive ponds, and usage of conventional energy resources, needs to be reduced. Future intensification of aquaculture techniques is only possible in accordance with existing regulations through improved spatial planning, in order to minimise negative impact, reduce emission and cope with the ongoing climate changes.

## CONCLUSIONS

Due to very different settings (geographical, meteorological, oceanographic), different preferences (consumer, market, traditional) and different ethnics and country-specific economies, the lessons learned differ from country to country. However, when south-south collaboration is more promoted (e.g., through close scientific collaboration, as it happens already since a few years), the three countries can benefit from each other’s experiences.

In Madagascar, a private company (instead of the government) took the lead and initiated commercial aquaculture with initially giving incentives (fry for free) to local communities to establish both seaweed and sea cucumber farming. Continued support through the first production cycles and reliable contracts to buy the harvest back were key elements. After ten years an evaluation resulted in the decision to continue this CBA scheme. Also, coral culture was introduced as additional commodity. It is concluded that several lessons have been learned and the CBA so far is a success story and could serve as a template for other coastal regions, also in other tropical countries. Future orientation should strongly support additional co-culture schemes, in an ideal case IMTA.

In Tanzania, the first steps into aquaculture were taken by private companies followed by women/organisations some 30 years ago, with the major focus to earn own income and become more independent from the husband. In a second step, it was planned to introduce co-culture with, e.g., sea cucumbers. But the lack of fry was identified as major bottleneck. Learning its lesson, recently the government working with KOICA and FAO and a local research institution State University of Zanzibar (SUZA) established a grant funded hatchery for sea cucumbers, crabs and milkfish, as potential IMTA add on’s to the meanwhile financially less attractive seaweed culture. Here improvements of the seaweed culture methods and adoption of the CBA model from Madagascar for co-culture could be the future. The recent joint efforts by scientists, government and NGOs definitely point into the right direction.

Indonesia is too big with too many different settings (climate, more than 17,000 small and large islands, ethnics, languages and extremes with regard to economic background of districts) in order to draw generalised conclusions. It has very long coastlines and a complicated legislation, therefore IMTA seems to be particularly suitable. In the last ten years the government followed up on two main lessons learned: the lack of brood-stock and fry availability for aquaculture, and the legislation mainly build from land-based perspectives with emphasis on agriculture. Therefore, a number of decentralised training and brood-stock centres were established all-over the country. And the new Ministry for Marine Affairs and Fisheries was established to separate agriculture from fisheries and aquaculture. In the meantime, IMTA was successfully tested in model regions – however streamlined political support from several ministries is urgently needed.
